# Supplementary material for: On a Cold Night: Transcriptomics of Grapevine Flower Unveils Signal Transduction and Impacted Metabolism
Source: Int J Mol Sci. 2019 Mar 5;20(5):1130. doi: 10.3390/ijms20051130 (PMC6429367; doi:10.3390/ijms20051130)
Supplement: Supplementary file 1 [file ijms-20-01130-s001.zip › ijms-453521-supplementary-3/supplementary file 1.docx]

**Supplementary file 1.** Genes, annotations, primer sequences used for real-time qPCR and CRIBI numbers.

| **Gene** | **Annotation** | **Sequences 5'-3'** | **CRIBI GGDB 12X V1 accession** |
| --- | --- | --- | --- |
|  |  |  |  |
| *EF1-α* | Elongation factor 1-alpha | AACCAAAATATCCGGAGTAAAAGA | VIT_06s0004g03240 |
|  |  | GAACTGGGTGCTTGATAGGC |  |
| *60* *RSP* | 60S ribosomal protein L18 | ATCTACCTCAAGCTCCTAGTC | VIT_05s0094g00870 |
|  |  | CAATCTTGTCCTCCTTTCCT |  |
| *PG* | Polygalacturonase | ACATCGGCAGTGGCTCTATC | VIT_01s0127g00400 |
|  |  | ATTGGAGGGACGGTAGGAGT |  |
| *TL1* | Thaumatin-like 1 | TGGCAAATGCGAAACTGGTG | VIT_13s0064g01310 |
|  |  | CCTTCAACTCACTGGGGCAT |  |
| *CBF* | CBF-like transcription factor | GTTTGCCCGTGCCAGTTTC | VIT_02s0025g04460 |
|  |  | TGATGCTTGATTCAGCAGCCT |  |
| *RS* | Raffinose synthase | CAGGCACGATGATGATCCCA | VIT_14s0066g00810 |
|  |  | CTTGGTAAGGGCAGTAGGGC |  |
| *PSIIp13* | Chlorophyll a/b-binding protein, LHCB3 | TGGGCTTCCAAGTCGTACTG | VIT_00s0181g00180 |
|  |  | GGTGACAGGGTCATCAGCAA |  |
| *4CL* | 4-coumarate-CoA ligase-like 1 | GCTCAAGCTTAGGGCAGTCA GCTGTGCTCAGTCAGTCCAT | VIT_01s0010g03720 |
| *STSa* | Stilbene synthase 5 | GGTCACTAAGAGCGAGCACA GCCTTCAATGCTGCTTCCTT | VIT_10s0042g00840 |
| *STS* | Stilbene synthase | AGGAAGCAGCATTGAAGGCTC  TGCACCAGGCATTTCTACACC | VIT_16s0100g00780 |
| *PR1* | pathogenesis-related protein 1 | GGAGTCCATTAGCACTCCTTTG | VIT_203s0088g00810 |
|  |  | CATAATTCTGGGCGTAGGCAG |  |
| *CHI4C* | Class IV chitinase | TCGAATGCGATGGTGGAAA  TCCCCTGTCGAAACACCAAG | VIT_05s0094g00360 |
| *LHCA4* | Chlorophyll a/b-binding protein, LHCA4 | CAACCCCCTGAACTTTGCAC | VIT_17s0000g06350 |
|  |  | GCCCTTTTCCAGTCACGTTG |  |
| *GIN1* | Vacuolar invertase 1 | GTCTTTGGCCGTGGATGATG | VIT_16s0022g00670 |
|  |  | GGCTAAAGCCTTCCACAATCG |  |
| *SUC27* | Sucrose transporter-like | GTTGAGTTCTTTGGCCGTGG  GACGCTGCCAACTTGCTAAC | VIT_18s0076g00250 |
