# Supplementary material for: On a Cold Night: Transcriptomics of Grapevine Flower Unveils Signal Transduction and Impacted Metabolism
Source: Int J Mol Sci. 2019 Mar 5;20(5):1130. doi: 10.3390/ijms20051130 (PMC6429367; doi:10.3390/ijms20051130)
Supplement: Supplementary file 1 [file ijms-20-01130-s001.zip › ijms-453521-supplementary-3/supplementary file 12.docx]

| 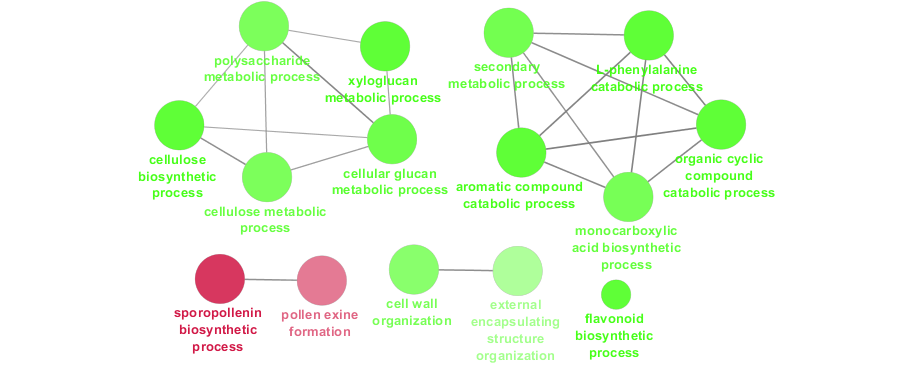 |
| --- |
| a |
| 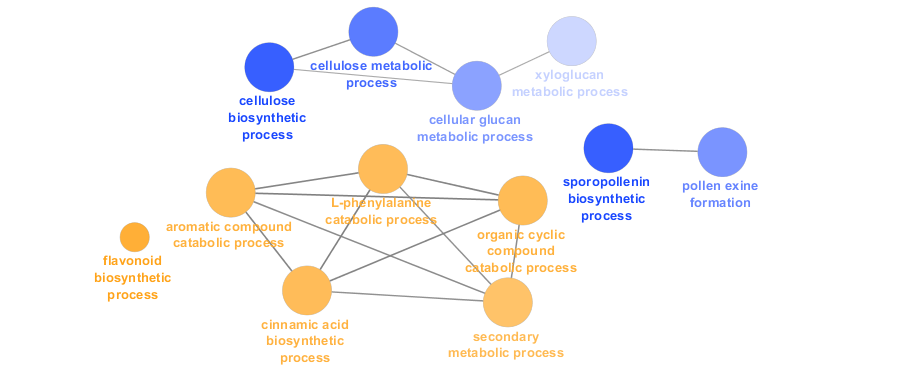 |
| b |

**Supplementary File 12:** GOE analysis visualized in Cytoscape with the ClueGO plugin showing in (**a**) the up- and down-regulated ontologies ad 2h (red and green, respectively) and in (**b**) those at 8h (blue for up-regulated and orange for down-regulated).
