# Supplementary figures and images for: On a Cold Night: Transcriptomics of Grapevine Flower Unveils Signal Transduction and Impacted Metabolism
Source: Int J Mol Sci. 2019 Mar 5;20(5):1130. doi: 10.3390/ijms20051130 (PMC6429367; doi:10.3390/ijms20051130)

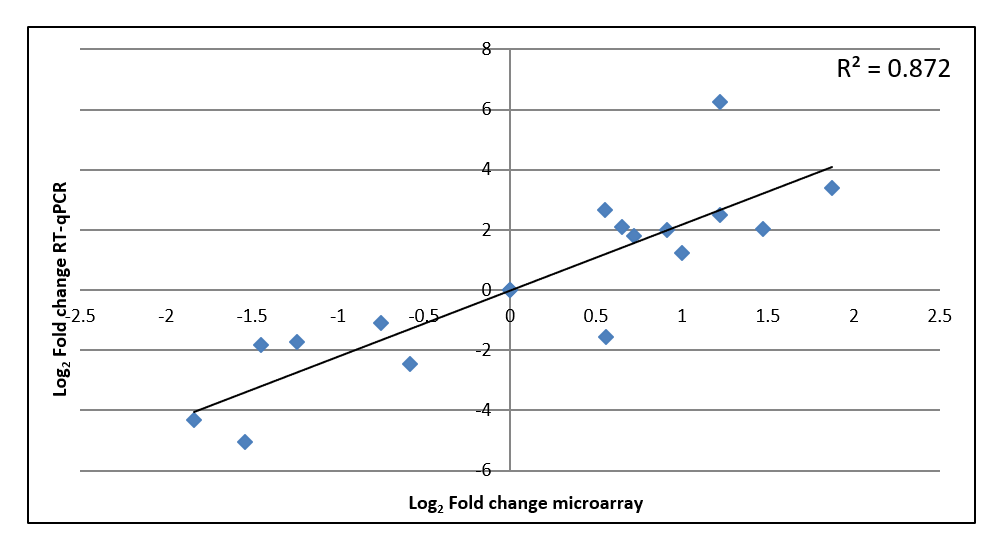


**Supplementary file 2.** Validation of the microarray results

Supplement: Supplementary file 1 [file ijms-20-01130-s001.zip › ijms-453521-supplementary-3/supplementary file 2.docx]
